# Study on Load-Bearing Characteristics of a New Pile Group Foundation for an Offshore Wind Turbine

**DOI:** 10.1155/2014/394104

**Published:** 2014-08-28

**Authors:** Ruiqing Lang, Run Liu, Jijian Lian, Hongyan Ding

**Affiliations:** State Key Laboratory of Hydraulic Engineering Simulation and Safety, Tianjin University, Tianjin 300072, China

## Abstract

Because offshore wind turbines are high-rise structures, they transfer large horizontal loads and moments to their foundations. One of the keys to designing a foundation is determining the sensitivities and laws affecting its load-bearing capacity. In this study, this procedure was carried out for a new high-rise cap pile group foundation adapted to the loading characteristics of offshore wind turbines. The sensitivities of influential factors affecting the bearing properties were determined using an orthogonal test. Through a combination of numerical simulations and model tests, the effects of the inclination angle, length, diameter, and number of side piles on the vertical bearing capacity, horizontal bearing capacity, and bending bearing capacity were determined. The results indicate that an increase in the inclination angle of the side piles will increase the vertical bearing capacity, horizontal bearing capacity, and bending bearing capacity. An increase in the length of the side piles will increase the vertical bearing capacity and bending bearing capacity. When the length of the side piles is close to the central pile, the increase is more apparent. Finally, increasing the number of piles will increase the horizontal bearing capacity; however, the growth rate is small because of the pile group effect.

## 1. Introduction

Offshore wind power, which is stable and sustainable, is a new type of energy resource. This resource can generate large amounts of power and does not require the support of land resources. Furthermore, wind power has been favored by several countries in recent years [1]. Among the factors that ensure offshore wind turbines operating safely and stably, the foundation is top priority. Currently, the types of foundations used for offshore wind turbines include large-diameter single pile, pile group, tripod, jacket, gravity base, bucket, and floating. The Donghai Bridge offshore wind farm is Asia's first large-scale offshore wind farm to use a high-rise cap pile group foundation successfully. Based on the large displacements at the top of the piles generated by a relatively small vertical load and a large horizontal load, as well as large moments produced by a horizontal force, which are also difficult to control, an offshore wind power research team in Tianjin University developed a new high-rise cap pile group foundation. The base consists of a large-diameter pile and several small diameter and length inclined piles. The inclined piles are evenly distributed around the large-diameter pile; this arrangement takes full advantage of the strong vertical bearing capacity of the large-diameter pile [2] and the strong horizontal bearing capacity and flexural capacity of the inclined piles. Moreover, the base can use the soil surrounding the central pile to bear loads effectively.

The new high-rise cap pile group foundation uses inclined piles, which have been widely used in bridge, wharf, and large transmission line foundations. The foundation's bearing capacity has become a highly popular research topic. Currently, several domestic and foreign researchers have studied the axial and horizontal operating performance of inclined piles. In a study on axial bearing capacity, Meyerhof et al. [3] observed that the axial bearing capacity of a batter pile increases with an increase in the inclination angle through analysis of field test data. Hanna and Nguyen [4] observed that the axial bearing capacity of the inclined pile decreases with an increase in the inclination angle by analyzing model test data. Hanna and Afram [5] believed that with an increase in the inclination angle, the axial bearing capacity does not change appreciably based on a further study on test data. Zheng et al. [6] concluded that the inclination angle has an effect on the settlement of the pile and the failure mode of the foundation under specific soil conditions and pile conditions through laboratory model tests combined with numerical simulations; in the researchers' study, an impact threshold value was determined to exist. In a study on horizontal bearing capacity, Zhang et al. [7] conducted a small-scale centrifuge model test of inclined piles and considered the effects of the inclination angle and the relative density of sand on the horizontal resistance of a single inclined pile. The results indicated that a negatively inclined pile has a greater horizontal resistance compared to that of a vertical pile and a positively inclined pile has a smaller horizontal bearing capacity compared to that of a vertical pile. Kavazanjian [8] determined that the inclined pile has an advantage in resisting horizontal forces. By summarizing the results of previous studies, Gerolymos et al. [9] demonstrated that a pile group foundation with a batter pile could provide greater horizontal stiffness. Yuan [10] conducted research on horizontal bearing capacity using a laboratory model test combined with theory analysis. Yuan proposed that the horizontal bearing capacity of a positively inclined pile is greater than that of a vertical pile and a negatively inclined pile; the negatively inclined pile was demonstrated to have the lowest capacity. The above-described findings provide good references regarding the application of inclined piles in the field of offshore wind power. However, there are no unified conclusions concerning bearing capacity for an inclined pile.

For the new high-rise cap pile group foundation considered in this report, the author studied the effects of different inclination angles of inclined piles on the vertical bearing capacity, horizontal bearing capacity, and flexural loading capacity and developed an optimal design for the new high-rise cap pile group foundation. The vertical and horizontal bearing capacities of the new high-rise cap pile group foundation were determined through laboratory model tests. The test results were analyzed using a numerical simulation method with the finite element analysis software program ABAQUS. The numerical simulation method provides the basis for the actual design of offshore wind power foundations.

## 2. Analysis of New High-Rise Cap Pile Group Foundation

### 2.1. Project Outline

**Figure 1:**
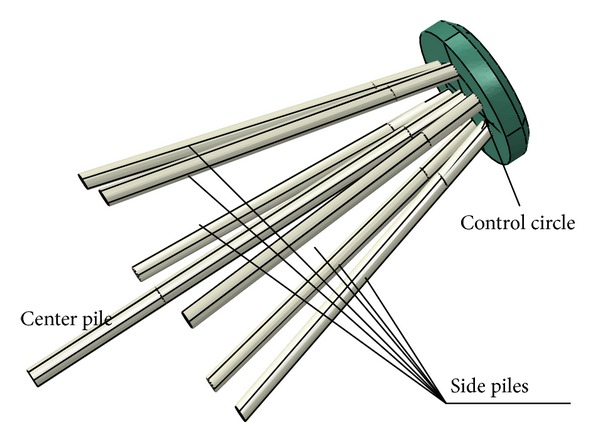
New type of pile group.

The foundation of a wind turbine, unlike other common structural foundations, must bear large moments. As indicated in Figure 1, the new high-rise cap pile group foundation proposed in this report consists of a large-diameter pile and several small diameter and length inclined piles. The inclined piles are evenly distributed along the control circle, the center of which is located at the center of the cap.

It is proposed that the vertical load is primarily supported by the central pile, whereas the horizontal force and bending moment are primarily supported by the side piles through the combination of a reasonable layout of the piles, a change in the inclination angle, and the length of the piles. Furthermore, the combination of the strong vertical bearing capacity of the large-diameter piles and the strong horizontal bearing capacity and flexural capacity of the inclined piles allows the foundation to control the horizontal displacement and discrepancy settlement, which are a result of the effectively large horizontal force and bending moment sustained by the foundation.

### 2.2. Analysis of Sensitive Dimensions for the Foundation

#### 2.2.1. Orthogonal Design Plan

There are several factors that affect the bearing capacity of the new pile group, such as the soil properties, pile length, distance between piles, number of piles, and shapes and sizes of the pile group.

To improve the bearing capacity of the foundation and determine the characteristic parameters of the bearing capacity effectively, an orthogonal experiment was performed to find the sensitivities of the various design parameters.

An orthogonal experiment using four factors and five levels was designed, which included 25 sets of tests. The four influential factors are the inclination angle of side piles Φ, the length of the side piles *L*, the diameter of the side piles *D*, and the number of piles *n*. The inclination angles of the side piles are 4°, 8°, 12°, 16°, and 20°. The lengths of the side piles are 15 m, 20 m, 25 m, 30 m, and 35 m. The diameters of the side piles are 0.8 m, 1.0 m, 1.2 m, 1.5 m, and 2.0 m, and the numbers of piles are 5, 6, 7, 8, and 9. The diameter of the central pile for all foundations is 2 m, and the length of the central pile is 40 m. The side piles are evenly distributed along the control circle, which has a radius of 6 m. The wall thickness of all piles is 30 mm.

#### 2.2.2. Implementation of Orthogonal Experiment

**Figure 2:**
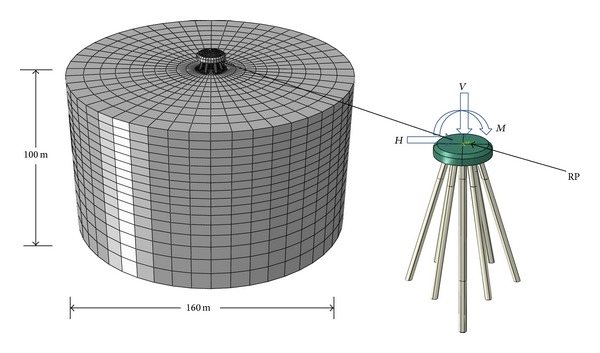
Loads on finite element model used in analyses.

A numerical simulation method was used to complete the orthogonal experiment. The numerical analysis model and the boundary conditions are provided in Figure 2.

To eliminate the side effect [11], a cylindrical soil (with diameter = 160 m and height = 100 m) was selected for numerical simulation. The foundation was assumed to be a perfectly elastic constitutive model made of steel. The density of steel, *ρ*, is 7850 kg/m^3^, Young's modulus, *E*, is 2.1 × 10^11^ Pa, and Poisson's ratio, *μ*, is 0.3. To study the effects of various design parameters on the bearing capacity and settlements of the new pile group foundation, the distribution of the soil layers was simplified. A homogenous soil was selected in the analysis. The Mohr-Coulomb failure criterion was applied to the soil material, and the hypothetical parameters of the soil are provided in Table 1. The tangential contact between the piles and the soil is frictional contact and is associated with a friction coefficient of 0.4, and the normal contact between the piles and the soil is hard contact. A tie is used to connect the piles and the cushion cap. When the model is complete, a vertical displacement load, a horizontal displacement load, and a bending moment load are applied at point RP, which is located at the top of the caps.

#### 2.2.3. Results of Orthogonal Experiment

The degrees of influence of the various factors on the vertical ultimate bearing capacity *V*_*u*_, horizontal ultimate bearing capacity *H*_*u*_, and flexural ultimate bearing capacity *M*_*u*_ were determined after analyzing the test data. The specific results are provided in Table 2.

As indicated in Table 2, for the new high-rise cap pile group foundation, *V*_*u*_ is primarily affected by the length of the side piles *L* and the inclination angle of the side piles Φ; *H*_*u*_ is primarily affected by the inclination angle of the side piles Φ and the number of side piles *n*; and *M*_*u*_ is primarily affected by the length of the side piles *L* and the inclination angle of the side piles Φ. To effectively improve the bearing capacity of this foundation, the length *L*, the inclination angle Φ, and the number of side piles *n* were studied independently.

### 2.3. Analysis of Influential Factors Affecting Bearing Capacity for New Pile Group

#### 2.3.1. Analysis of Inclination Angle of Side Piles

**Figure 3:**
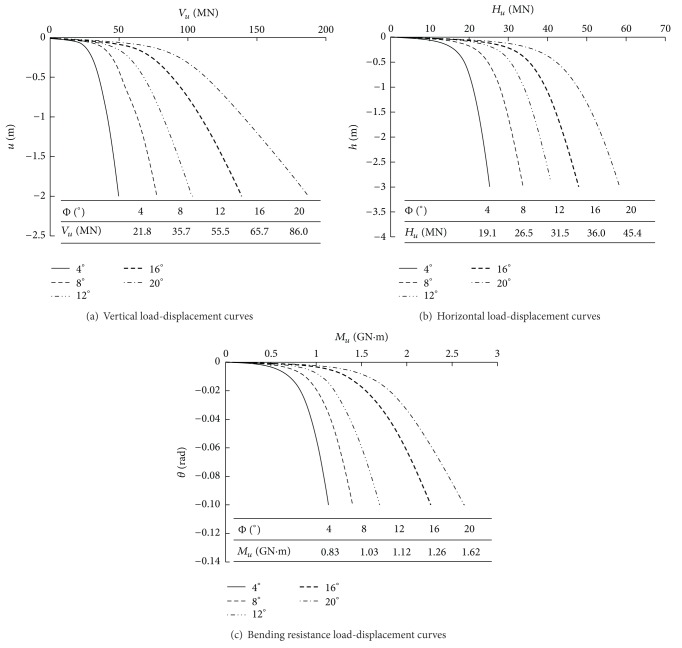
Load-settlement curves and ultimate bearing capacity of pile groups with different degrees of inclination.

Five different values of Φ were studied: 4°, 8°, 12°, 16°, and 20°. Other factors of the foundation remained unchanged. To study the influence of the inclination angle of the side piles on the bearing capacity under a single force, *V*, *H*, or *M* were applied to the foundation, respectively. The load-displacement curves and ultimate bearing capacity are presented in Figure 3. In this figure, *u* indicates vertical displacement, *h* indicates horizontal displacement, and *θ* indicates angle displacement.

As indicated in Figure 3, for pile groups with different inclination angles of side piles, the relationship between the load and the displacement of the foundation is linear when the load is small, and changes between the pile groups are similar. With an increase in load, the displacement of the pile group increases rapidly, and an inflection point appears. The greater the Φ is, the greater the load that corresponds to the inflection point becomes, which indicates that there is a positive correlation between the ultimate bearing capacity of the pile group and Φ. With Φ = 4° as a benchmark, when Φ is 8°, 12°, 16°, and 20°, *V*_*u*_ increases by 63%, 154%, 201%, and 294%, respectively; *H*_*u*_ increases by 39%, 65%, 88%, and 138%, respectively; and *M*_*u*_ increases by 24%, 35%, 52%, and 95%, respectively.

#### 2.3.2. Analysis of Length of Side Pile

**Figure 4:**
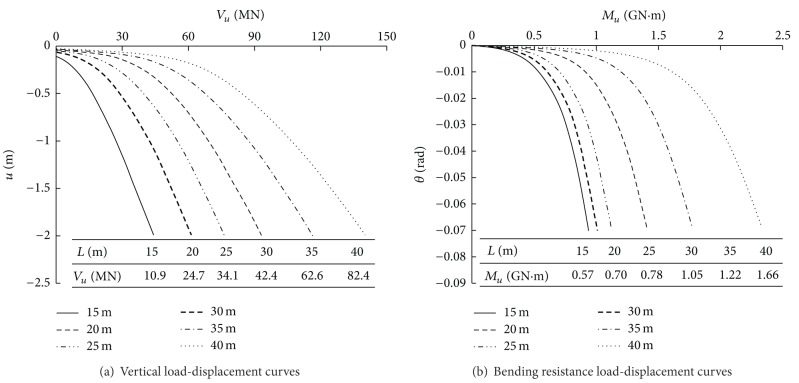
Load-settlement curves and ultimate bearing capacity of pile group with different side pile lengths.

Six different values of *L* were studied: 15 m, 20 m, 25 m, 30 m, 35 m, and 40 m. Other properties of the foundation remained constant. To study the influence of the length of the side piles on the bearing capacity under a single force, *V* or *M* was applied to the foundation, respectively. The resulting load-displacement curves and ultimate bearing capacity are presented in Figure 4.

As indicated in Figure 4, for pile groups with short side piles, there is a large settlement when the load is insignificant. For pile groups with long side piles, the relationship between the load and the displacement of the foundation is linear when the load is small, and changes between the pile groups are similar. With an increase in the load, the displacement of the pile groups increases rapidly, and an inflection point appears. In this case, the greater the value of *L* is, the greater the load that corresponds to the inflection point becomes, which indicates that there is a positive correlation between the ultimate bearing capacity and *L*. With *L* = 15 m as a benchmark, when *L* is 20 m, 25 m, 30 m, 35 m, and 40 m, *V*_*u*_ increases by 126.6%, 212.8%, 289.0%, 474.3%, and 656.0%, respectively; and *M*_*u*_ increases by 18%, 37%, 84%, 114%, and 191%, respectively.

#### 2.3.3. Analysis of the Number of Piles

**Figure 5:**
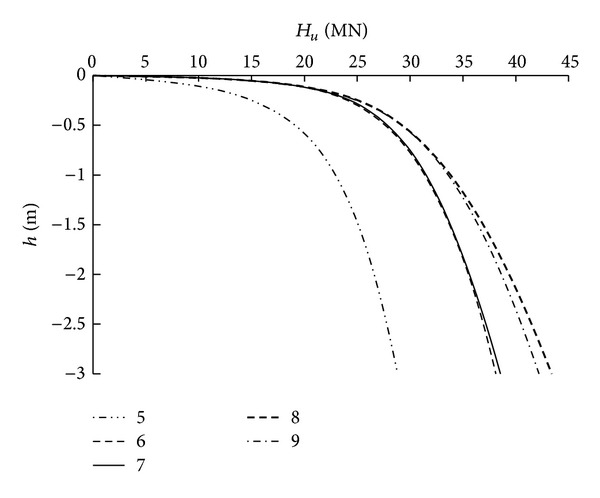
Load-settlement curves of pile group with different numbers of piles.

Five different values of *n* were studied: 5, 6, 7, 8, and 9. Other properties of the foundation remained constant. To study the influence of *n* on the bearing capacity under a single force, *H* was applied to the foundation. The corresponding load-displacement curves and ultimate bearing capacity are presented in Figure 5.

As indicated in Figure 5, for pile groups with different *n*, the relationship between the load and the displacement of the foundation is linear when the load is small, and changes between the pile groups are similar. With an increase in the load, the displacement of the pile group increases rapidly, and an inflection point appears. The changes in displacement between the pile groups with *n* = 6 and 7 are similar as are those between the pile groups with *n* = 8 and 9. As a reference, inflection points were selected to determine the ultimate bearing capacity. The horizontal bearing capacities were 19.3, 23.2, 23.9, 25.4, and 24.6 MN. With *n* = 5 as a benchmark, when *n* is 6, 7, 8, and 9, *H*_*u*_ is increases by 20.2%, 23.8%, 31.6%, and 27.5%, respectively.

Thus, with an increase in *n*, the horizontal bearing capacity of the pile group foundation increases. The effect of the horizontal bearing capacity is weak when *n* is greater than or equal to 6. The effect of the pile group is the main cause of this phenomenon.

## 3. Verification with Model Test

The author conducted a laboratory model test using a model of the new high-rise pile group foundation. Two types of bearing capacity were studied: vertical bearing capacity and horizontal bearing capacity. Furthermore, a numerical simulation method was used to simulate the laboratory model test, and the applicability of the numerical simulation was verified.

### 3.1. Laboratory Model Test

**Figure 6:**
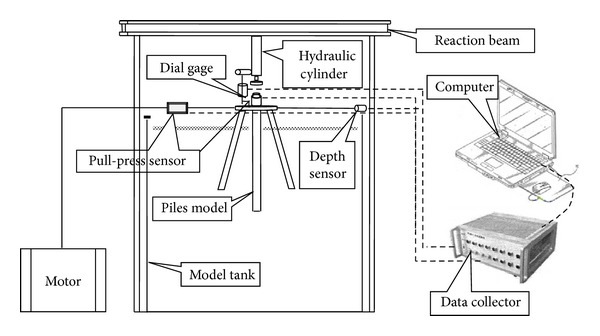
Map of model test apparatus.

As indicated in Figure 6, the model test apparatus consists of three parts: a model tank, a loading system, and a data capture system. The net dimensions of the model tank are 1.2 m × 1.2 m × 1.5 m. The loading system is divided into a vertical hydraulic loading system and a horizontal motor loading system. The vertical hydraulic loading device consists of a three-phase induction motor and a single-acting hydraulic cylinder. The working stroke length of the hydraulic cylinder is 250 mm, and the nominal tonnage is 20 t. The horizontal motor loading system consists of a frequency-variable three-phase asynchronous motor, pulley, and wire ropes. The motor is controlled by a frequency conversion inverter. The horizontal speed of the wire rope is 18 mm/min. The data capture system consists of sensors and a collector. The sensors include pull-press sensors, a dial gage for displacement, and a depth sensor. The measurement range of the pull-press sensors is 30 kN; the measurement accuracy of the dial gage is 0.01 mm with a range of 50 mm; and the measurement accuracy of the depth sensor is 1 mm with a range of 750 mm. A static and dynamic strain gauge is used to collect data.

**Figure 7:**
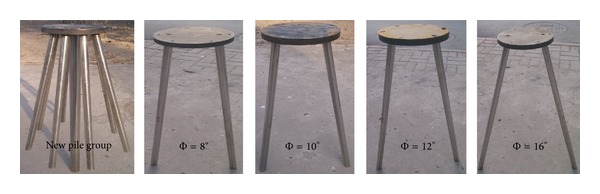
Test models.

The testing sand was sea sand, which was filled in the model tank in a stratified manner. The specific gravity of the soil grain was 2.67, and its density was 1.60 g/cm^3^. The thickness of the soil was 1.5 m. The foundation model consisted of a seamless steel pipe. A weld was used to connect the piles and cap, as indicated in Figure 7.

Two types of experiments were performed: the first experiment analyzed the values of *V*_*u*_ and *H*_*u*_ of the new high-rise cap pile group foundation and the other experiment examined the influence of Φ on *V*_*u*_ and *H*_*u*_, and models for the two symmetrically inclined piles were selected in this experiment. The new high-rise cap pile group foundation (length of the central pile was 54.3 m, *L* = 40 m, *n* = 9, Φ = 8°, and *D* = 2 m) was selected as the prototype of the foundation model. Furthermore, the model featured a scaled-down ratio of 1 : 100. The scaled-down parameters included the diameter of the piles, the length of the piles, and the size of the cap. Due to the limits associated with the processing materials, only the wall thickness, which has little effect on the bearing capacity, was not scaled down completely to 1 : 100. For the models of the two symmetrically inclined piles (*L* = 40 cm), different Φ values were studied: 8°, 10°, 12°, and 16°. The values of 8°, 10°, 12°, and 16° were selected to study the influence of Φ on *V*_*u*_. The values of 8°, 12°, and 16° were selected to study the influence of Φ on *H*_*u*_.

### 3.2. Analysis of Test Data

#### 3.2.1. Analysis of Vertical Results

**Figure 8:**
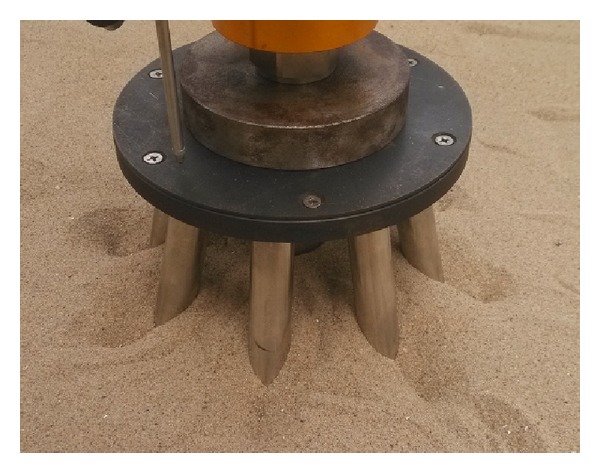
Deformation of the soil surface.

**Figure 9:**
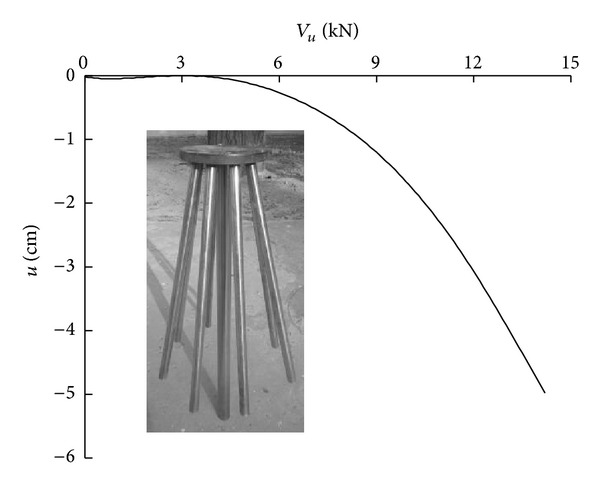
Load-settlement curve of new pile group.

The deformation of the soil surface observed after loading was complete is shown in Figure 8. The load-displacement curve of the foundation model is presented in Figure 9.

As indicated in Figure 8, a local subsidence appears on the surface of the soil surrounding each of the side piles, covering an area measuring three times smaller than the diameter of a single pile. No large bulge appears on the surface.

As indicated in Figure 9, the relationship between the load and the displacement of the foundation is linear when the load is small. With an increase in the load, the slope of the load-displacement curve increases rapidly, and an inflection point appears. The vertical bearing capacity of the foundation model is 7.2 kN.

#### 3.2.2. Analysis of Horizontal Results

**Figure 10:**
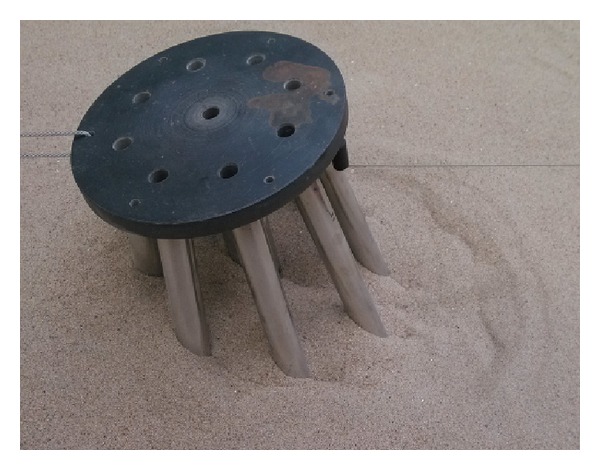
Deformation of the soil surface.

**Figure 11:**
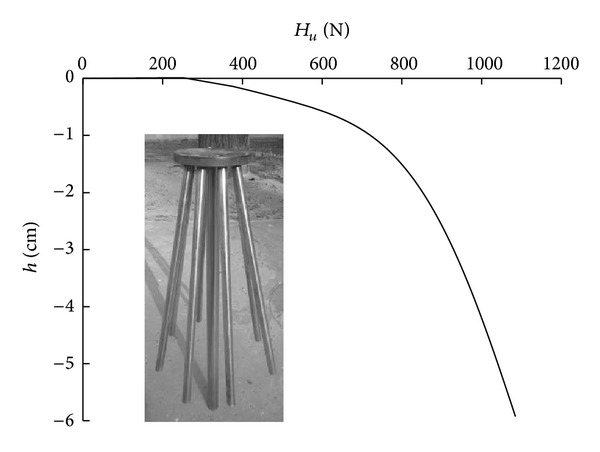
Load-settlement curve of new pile group.

The deformation of the soil surface after loading is complete and is presented in Figure 10. The load-displacement curve of the foundation model is provided in Figure 11.

As indicated in Figure 10, a complete depression appears behind the model, and the model is locally inclined, which indicates that the local soil is structurally failing under the horizontal force.

As indicated in Figure 11, the relationship between the load and the displacement of the foundation is linear when the load is small. With an increase in the load, the slope of the load-displacement curve increases rapidly, and an inflection point appears. The horizontal bearing capacity of the foundation model is 701 N.

#### 3.2.3. Analysis of Test Results for Influence of Inclination on Vertical Bearing Capacity

**Figure 12:**
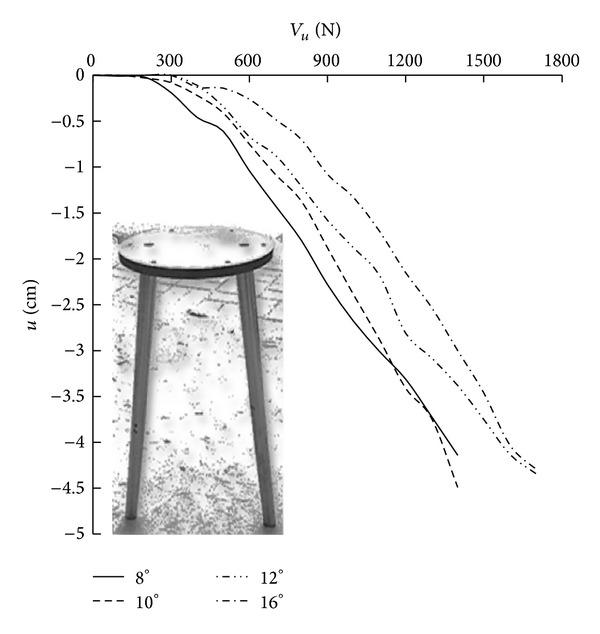
Load-settlement curves of inclined piles with different degrees of inclination.

When the vertical load force at the center of the four models with different Φ values, which consisted of two symmetrically inclined piles, was complete, test data were collected. The load-displacement curve of the models is provided in Figure 12.

As indicated in Figure 12, the relationship between the load and the displacement of the foundation is linear when the load is small. With an increase in the load, the slope of the load-displacement curve increases rapidly, and an inflection point appears. After a further analysis of the load-displacement in Figure 12, it is determined that, under the same load, an increase in the inclination angle of piles results in a decrease in the settlement of the corresponding model. As a reference, inflection points are selected to determine the ultimate bearing capacity. The vertical bearing capacities for the inclined piles with different Φ values are 450, 500, 600, and 710 N. With Φ = 8° as a benchmark, when Φ is 10°, 12°, and 16°, *V*_*u*_ increases by 11%, 33%, and 57%, respectively. Overall, with an increase in Φ, the corresponding ultimate bearing capacity of the foundation increases. This conclusion is consistent with the analysis of factors that affect bearing capacity.

### 3.3. Verification of Numerical Simulation Method

**Figure 13:**
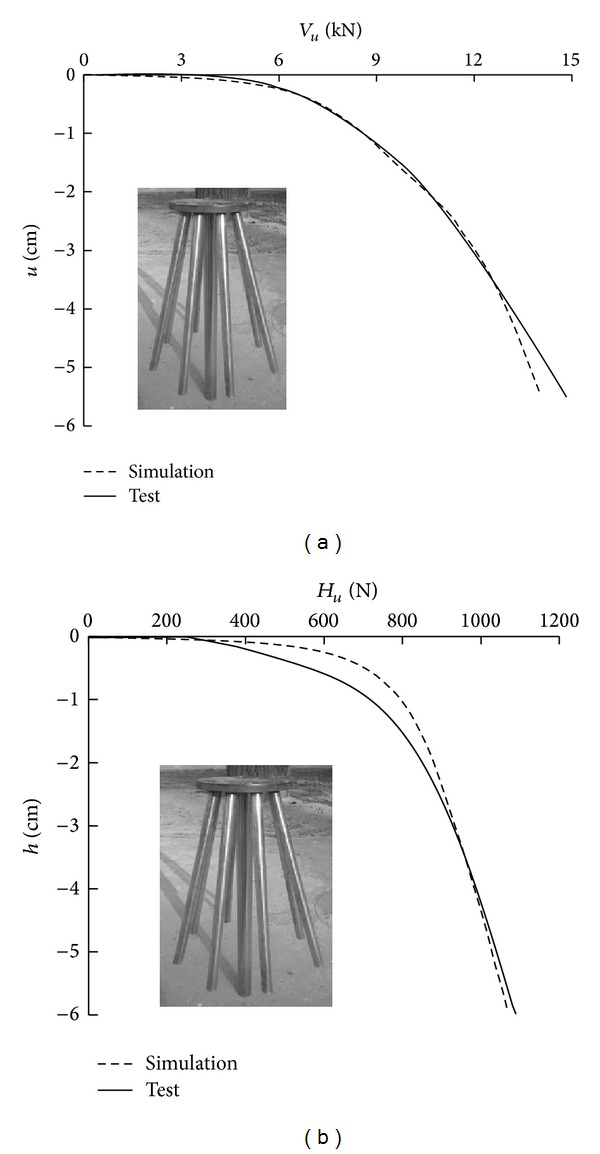
Load-settlement curves of new type of pile group.

Based on the actual size of the laboratory model test, the new pile group experiment was simulated. The Mohr-Coulomb failure criterion was applied to the soil. The unit weight was 16.0 kN/m^3^, Poisson's ratio was 0.3, the elasticity modulus was 24 MPa, the internal friction angle was 32°, and the cohesion was 2 kPa.  Figure 13 compares the test and simulated load-displacement curves of the new high-rise cap pile group foundation.

As indicated in Figure 13, the simulation curves and the measured curves are relatively similar. Inflection points appear in both of these curves. The vertical bearing capacity of the simulated system is 8.08 kN, whereas that for the test system is 7.2 kN. The horizontal bearing capacity of the simulated system is 790 N, whereas that for the test system is 701 N.

**Figure 14:**
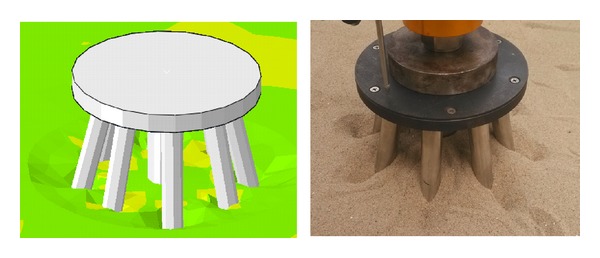
Comparison between test deformation and FEM results for vertical loading of pile group.

Figure 14 compares the deformation of soil under a single vertical load observed in the numerical simulation with the test deformation.

As indicated in Figure 14, the deformation in the numerical simulation and test deformation are relatively similar. The surface of the soil sinks near the side piles when the vertical loading is complete. The sinking of the soil affects the soil nearby and forms a local subsidence. However, there is no integral settlement throughout the soil. A significant stress concentration appears in the area of contact and spreads to the surrounding soil.

**Figure 15:**
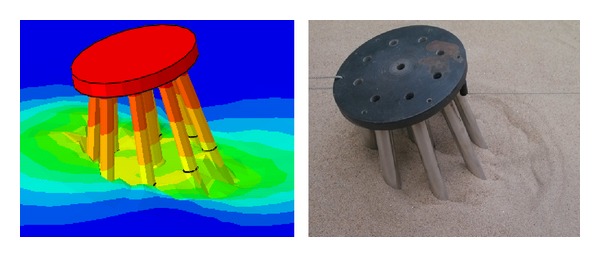
Comparison between test deformation and FEM results for lateral loading of pile group.

Figure 15 compares the deformation of soil under a single horizontal load applied in the numerical simulation with the test deformation.

As indicated in Figure 15, the deformation in the numerical simulation and the test deformation are relatively similar. Significant traces of movement of the piles appear, which causes the soil near the piles to deform. However, the effective range of deformation is limited, and the local soil behind the pile group is in structural failure. The pile group is locally inclined, which demonstrates that the soil is completely destroyed under the horizontal load.

In conclusion, the numerical simulation method established in this report is applicable to the simulation of new high-rise cap pile group foundations.

## 4. Example Project 

### 4.1. General Engineering Information

An offshore wind power farm is proposed to be constructed. According to a geological survey of the seabed, the geotechnical spatial distribution is complex. From top to bottom, the seabed can be divided into 11 layers within the surveying depth. Several of these layers are soft soil or hard soil. The specific soil parameters are provided in Table 3.

The load cases for 3 MW wind turbines are shown in Table 4. Load Case A is used to verify the bearing capacity of the foundation. Load Case B is used to verify the antioverturning stability and antisliding stability of the foundation.

### 4.2. Design and Analysis

In the design of the foundation structure, different load effect combinations were selected to calculate different design content. A foundation featuring one central pile and six side piles was designed. The six side piles were evenly distributed along the control circle. The diameter of the central pile was 3 m, and its total length and buried depth were 67.5 m and 50 m, respectively. The diameter of the side pile was 1.5 m, and the total side pile length and buried depth were 60 m and 42.5 m, respectively. The diameter of the control circle was 10.4 m. The side piles inclined outwardly along a line that connected the center of the cap and the center of the central pile. The rake ratio of the side piles was 1 : 6.

To verify the feasibility and safety of this foundation, the proposed numerical simulation method was performed. In the simulation, the load cases shown in Table 4 were loaded at the center of the top of the cap simultaneously. The bearing capacities of the foundation, deformation, strength of the pile, and other parameters were acquired through calculation.

**Figure 16:**
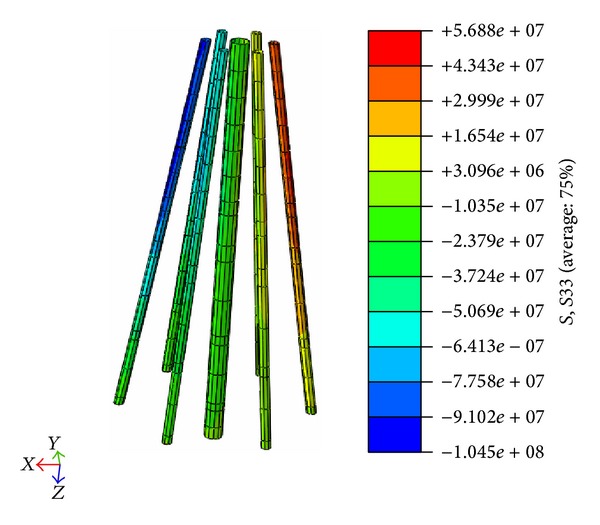
Tension and compression stress distribution in the pile.

The tension and compression stress distributions under Load Case A are presented in Figure 16.

Based on FEM postprocessing, the maximum compression stress was determined to be 9604.9 kN, and the maximum tension stress was determined to be 3752.1 kN. Incorporating a structure importance coefficient of 1.1 into the calculation, the maximum compression stress was calculated to be 10565.4 kN, which is less than the value of 10757.1 MPa calculated by the Code for Pile Foundation in Harbor Engineering. The maximum tension stress was determined to be 4127.3 kN, which is less than the value of 5493.3 MPa calculated by the Code for Pile Foundation in Harbor Engineering.

**Figure 17:**
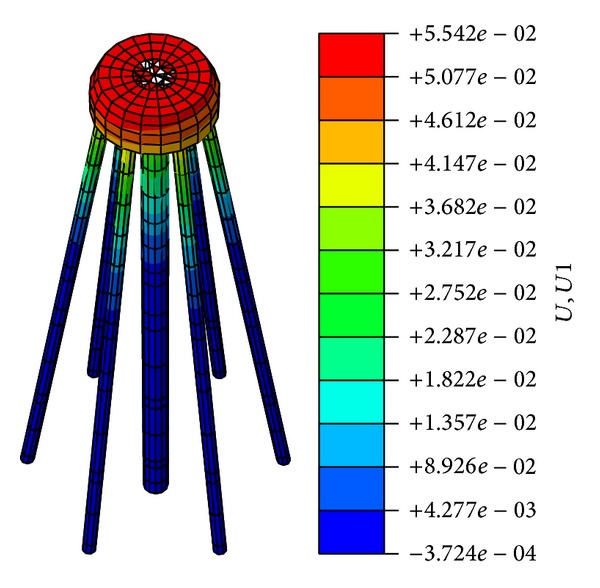
Horizontal displacement of foundation.

**Figure 18:**
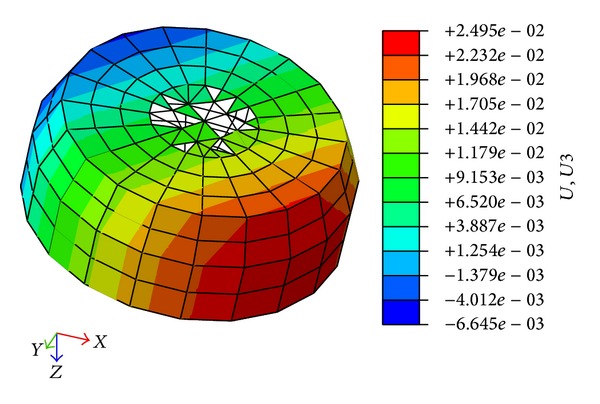
Vertical displacement of cap.

The horizontal displacement of the foundation and the vertical displacement of the cap are presented in Figures 17 and 18, respectively.

Figure 17 indicates that the maximum horizontal displacement is 55.42 mm.  Figure 18 indicates that the maximum downward vertical displacement is 24.95 mm, whereas the maximum upward vertical displacement is 6.65 mm. Thus, the differential settlement ratio is 2.2‰, which is less than 3‰. The safety factor of antioverturning is 4.27, which is greater than 1.3. The safety factor of antisliding is 3.96, which is greater than 1.6.

Thus, all of the results obtained meet the specified requirements.

## 5. Conclusion

Based on the characteristics of offshore wind loads, a new high-rise cap pile group foundation consisting of large-diameter piles and inclined piles was proposed, which takes full advantage of the strong vertical bearing capacity of the large-diameter piles and the strong horizontal bearing capacity and flexural capacity of the inclined piles. The influence of certain factors on the vertical bearing capacity, horizontal bearing capacity, and bending bearing capacity was studied through laboratory experiments and a numerical simulation method. The following conclusions can be drawn.

1. The primary factors affecting the bearing capacity of the new high-rise cap pile group foundation were determined using an orthogonal numerical simulation test. The primary factors affecting the vertical bearing capacity are the inclination angle and length of the side piles. For the horizontal bearing capacity, the primary factors are the inclination angle and number of piles. For the bending bearing capacity, the primary factors are the length and inclination angle of the side piles.
2. A numerical simulation method was conducted to study the primary factors affecting the bearing capacity. The results were verified by laboratory experiments. An increase in the inclination angle of the side piles increases the vertical bearing capacity, the horizontal bearing capacity, and the bending bearing capacity. An increase in the length of the side piles increases the vertical bearing capacity and the bending bearing capacity. When the length of the side piles is close to the length of the central pile, the increase in the bearing capacity is more apparent. Increasing the number of piles increases the horizontal bearing capacity; however, the rate of growth is small because of the pile group effect.
3. A foundation consisting of one central pile (*D* = 3 m, *L* = 67.5 m) and six side piles (*D* = 1.5 m, *L* = 60 m) with a rake ratio of 1 : 6 was designed for an offshore wind power project. The bearing capacity, deformation, and other important parameters under different load cases were investigated. All parameters were observed to meet the specified requirements. Thus, overall, the new high-rise cap pile group foundation is a suitable foundation for offshore wind turbines.

## Figures and Tables

**Table 1:** Parameters of soils in FEM simulation.

Unit weight *γ*/(kN/m^3^)	Poisson's ratio *μ*	Elastic modulus *E*/(MPa)	Internal friction angle *φ*/(°)	Cohesion *c*/(kPa)
7.0	0.3	30.0	0	10.0

**Table 2:** Influence of factors on bearing capacity for different directions.

Bearing capacity	Sensitive degree			
	Higher	High	Middle	Low
*V*_*u*_	*L*	Φ	*n*	*D*
*H*_*u*_	Φ	*n*	*L*	*D*
*M*_*u*_	*L*	Φ	*D*	*n*

**Table 3:** Soil parameters.

Stratum	Soil description	Thickness *h* (m)	Wet weight *γ* (kN/m^3^)	Compression modulus *Es*_0.1-0.2_ (MPa)	Consolidated quickly shear test	
					Cohesion *c* (kPa)	Internal friction angle *φ* (°)
1	Sludge	8.5	17.0	2.49	11.4	11.3
2	Clay	2.3	18.5	3.22	25.0	10.4
3	Silty sand	5.2	20	15	0	33
4	Silty clay	2.8	19.5	5.18	24.3	14.1
5	Silty sand	6.7	20	18	0	37
6	Silty clay	6.2	20.3	6.03	33.8	10.5
7	Silt	1.6	20.5	6.76	30.7	18.8
8	Silt	10.4	19.3	7.90	17.0	25.8
9	Silt	4.3	19.3	7.90	17.0	25.8
10	Silty clay	2	20.0	5.25	23.1	16.3
11	Silty sand	10	20	21	0	38

**Table 4:** Load cases.

Load case	*F*_*V*_ (kN)	*F*_*H*_ (kN)	*M* (kN*·*m)	*T* (kN*·*m)
Load case A	4147.0	4688.2	91524.26	3280.3
Load case B	5153.1	5598.5	126249.76	4428.4
